# Hypotension Prediction Index and Incidence of Perioperative Hypotension: A Single-Center Propensity-Score-Matched Analysis

**DOI:** 10.3390/jcm12175479

**Published:** 2023-08-23

**Authors:** Julian Runge, Jessica Graw, Carla D. Grundmann, Thomas Komanek, Jan M. Wischermann, Ulrich H. Frey

**Affiliations:** Department of Anaesthesiology, Operative Intensive Care Medicine, Pain and Palliative Medicine, Marien Hospital Herne, Ruhr-University Bochum, Hölkeskampring 40, D-44625 Herne, Germany

**Keywords:** hemodynamics, hypotension, monitoring, propensity score matching

## Abstract

(1) Background: Intraoperative hypotension is common and is associated with increased morbidity and mortality. The Hypotension Prediction Index (HPI) is an advancement of arterial waveform analysis and allows preventive treatments. We used a propensity-score-matched study design to test whether application of the HPI reduces hypotensive events in non-cardiac surgery patients; (2) Methods: 769 patients were selected for propensity score matching. After matching, both HPI and non-HPI groups together comprised *n* = 136 patients. A goal-directed treatment protocol was applied in both groups. The primary endpoint was the incidence and duration of hypotensive events defined as MAP < 65 mmHg, evaluated by the time-weighted average (TWA) of hypotension. (3) Results: The median TWA of hypotension below 65 mmHg in the matched cohort was 0.180 mmHg (IQR 0.060, 0.410) in the non-HPI group vs. 0.070 mmHg (IQR 0.020, 0.240) in the HPI group (*p* < 0.001). TWA was higher in patients with ASA classification III/IV (0.170 mmHg; IQR 0.035, 0.365) than in patients with ASA status II (0.100; IQR 0.020, 0.250; *p* = 0.02). Stratification by intervention group showed no differences in the HPI group while TWA values in the non-HPI group were more than twice as high in patients with ASA status III/IV (*p* = 0.01); (4) Conclusions: HPI reduces intraoperative hypotension in a matched cohort seen for TWA below 65 mmHg and relative time in hypotension. In addition, non-HPI patients with ASA status III/IV showed a higher TWA compared with HPI-patients, indicating an advantageous effect of using HPI in patients at higher risk.

## 1. Introduction

Intraoperative hypotension is common during noncardiac surgery [1]. The incidence of intraoperative hypotension varies widely but most patients experience at least one episode during surgery with a decrease of mean arterial pressure (MAP) to less than 65 mmHg [2]. Several data from retrospective studies have shown an association of intraoperative hypotension with increased incidence of myocardial infarction, renal insufficiency, and increased mortality [3,4]. In addition, these organ injuries have been shown to correspond to the duration, frequency and magnitude of the drop in mean arterial pressure, while a threshold of 65 mmHg appears to prevent myocardial and renal damage [5,6] and prevention of intraoperative hypotension has been reported to reduce the risk of organ dysfunction [7]. Furthermore, it has been shown that more severe comorbidities increase the risk of perioperative hypotension and postoperative complications [8]. However, blood pressure management is primarily reactive.

The Hypotension Prediction Index algorithm (HPI, Edwards Lifescience Corp., Irvine, CA, USA) was developed to predict hypotension, defined as a MAP that is less than 65 mmHg. It uses patient demographics—such as age, height, weight—and radial artery waveform analysis as a further development of the FloTrac algorithm based on a proprietary, machine learning system and ranges from 0 to 100; thus, it is able to predict hypotension 5 min in advance with 92% sensitivity and specificity when the index exceeds a value of 85 [9,10].The system also provides advanced hemodynamic information including cardiac output, dynamic arterial elastance, dP/dtmax (systolic slope), and stroke volume—all of which presumably helps clinicians select optimal treatments without recommending any particular treatment.

In this way HPI allows healthcare providers to proactively manage patients who are at high risk, with the aim of reducing the risk of adverse outcomes associated with intraoperative hypotension.

We have recently shown that the HPI was able to reduce incidence and duration of hypotension when compared with arterial waveform analysis alone [11]. However, a randomized controlled trial did not show a reduction in intraoperative hypotension episodes under application of the HPI. The authors discussed an impact of study design deriving from the fact that both HPI-using and non-HPI-using clinicians were asked to avoid MAPs below 65 mmHg. This is called the Hawthorne effect and the actual effects could thus remain masked [12]. While post-hoc analysis often reflects more real-life conditions, retrospective study designs often suffer from confounders which cannot be entirely eliminated. It is thus important to clarify the effects of the application of the HPI when treatment groups are comparable to give an answer to the inconsistent data presented in prior studies. Therefore, in order to incorporate more reliable data at the population level, we employed propensity score matching in the current work. This method allows the estimation of causal intervention effects in non-randomized research by minimizing the selection bias between interventional groups [13]. We anticipated that, by using this method, we would have a dataset with low bias as well as significant demographic, anesthetic and surgical information.

We used this study design to test the hypothesis that the additional application of the HPI in comparison with invasive blood pressure measurements alone reduces the incidence, duration and severity of hypotensive events in non-cardiac surgical patients.

## 2. Materials and Methods

This cohort study is based on information from our registry on perioperative hypotension in patients with invasive blood pressure measurements. Our registry provides details of each surgical procedure, including anesthetics used, and covers the full perioperative process from preoperative workup through discharge from the intensive care unit. Patients undergoing major surgery with a duration >30 min and receiving an arterial catheter between 1/2021 and 10/2022 were identified in the electronical database (patient data management system, PDMS) after receiving ethical approval (Ethical Review Board, Bochum, Germany; approval no. 20-7027-NIS). 

We included all datasets that presented complete preoperative, intraoperative and postoperative data from patients undergoing moderate- or high-risk non-cardiac surgery in the departments of urology, general surgery, vascular surgery, and gynecology. General anesthesia was performed either as balanced anesthesia or total-intravenous anesthesia. Patients undergoing major abdominal procedures were additionally treated with peridural anesthesia, taking into account any contraindications the patient may have. The intraoperative dose was administered by a bolus of 0.75% ropivacaine. Postoperative analgesia was provided by continuous application of 0.2% ropivacaine.

Baseline parameters before induction were taken from non-invasive blood pressure measurements before induction of anesthesia. All further data for blood pressure measurements were extracted from invasive-blood pressure monitoring via the inserted arterial catheter.

Each patient was treated according to an in-house standard protocol to maintain MAP above 65 mmHg or higher in patients with appropriate comorbidities to account for autoregulatory mechanisms. For all patients treated with hemodynamic monitoring using the HPI a goal-directed treatment (GDT) protocol was established to ensure standardized but individualized treatment. The GDT contained crystalloid fluids and vasopressors; in addition, colloids and inotropic medication were applied upon request of the clinician. Anesthetists in this cohort of patients were unaware of the intention to use the data for this study.

The decision to use HPI was based on the patient’s individual risk profile, but also on the planned procedure, the length of the procedure, or the expected blood loss. Furthermore, the use of the HPI was also influenced by low device availability compared with the center’s operating room capacity.

The presented data were extracted via the Philips IntelliSpace and Critical Care and Anesthesia reporting system (ICCA, Philips Medical Systems, Andover, MA, USA). For each patient a full dataset was generated via the SQL-database in the environment of Microsoft SQL Server Management Studio (version 18.7.1, Microsoft, Redmond, Washington, DC, USA). In cases of applied monitoring with HPI these additional data were transferred via an EC10 adapter to the Philips monitoring system and in this way stored in the ICCA system and made available via SQL-query. The data intervals for all submitted data were 30 s.

For the evaluation of perioperative hypotension, we calculated the time-weighted average (TWA) of hypotension for each patient. The TWA is defined as the intensity and length of the hypotensive episodes in relation to the overall operation or monitoring time, respectively. It is calculated by dividing the total area under the threshold by the total duration of the monitoring time. Therefore, it represents a combination of the severity and duration of hypotensive events in relation to the total monitoring time depending on the selected threshold, e.g., 65 mmHg.

A propensity score was used to match the monitoring groups (HPI- or non-HPI monitoring). The propensity score represents the likelihood that an intervention will be assigned based on the current baseline parameters. After the sample was established, the treatment effect could be calculated by directly comparing the groups’ results.

Categorical data are presented as frequencies with percentages. Differences were analyzed with the Fisher’s exact test. For quantitative variables, we tested for normal distribution using the Shapiro–Wilk test. Data are presented as means and standard deviation (SD) in case of normal distribution, otherwise, data are presented as median and interquartile range (IQR). Linear variables were analyzed using the Student’s unpaired *t*-test in normal distributed variables and the Mann–Whitney U-test in non-normally distributed variables.

For comparison of cohorts, we first analyzed the unmatched data which show demographical, perioperative and hemodynamic data for all *n* = 769 patients included in this study. Here, standardized mean differences were shown between the HPI- and the non-HPI-groups. Demographical and intraoperative variables (Table 1 and Table 2) were considered real confounders of the incidence of intraoperative hypotension as they were anticipated to be related to both the choice of monitoring method and intraoperative hypotension. These identified variables were used to create a propensity-score-matched cohort using the monitoring method (HPI vs. arterial wave form analysis) as the independent variable. For matching we included all variables listed in Table 1 and Table 2 as dependent/control variables.

All individual propensity scores were calculated through logistic regression models, and then the 1:1 nearest-neighbor propensity score matching with a caliper size of 0.1 was used. We also changed the caliper size (from 0.1 to 0.5) to test the robustness of the findings for the propensity-score-matched cohort. After this matching, each group (HPI and non-HPI) comprised *n* = 136 patients. All (absolute) standardized mean differences now had values below 0.2.

The primary outcome was the time-weighted average (TWA) of hypotension below 65 mmHg between HPI and non-HPI monitored subjects. Secondary endpoints were the number and cumulative duration of intraoperative hypotension as well as the time-weighted average of hypotension below 60 mmHg and 55 mmHg, respectively. We also presented the total of HPI events >85 in patients monitored with the HPI system. Furthermore, we classified all patients according to the WHO definition of acute kidney injury. For this purpose, we examined increases in serum creatinine of more than 0.3 mg/dL within 48 h post-surgery or increases in serum creatinine to 1.5 times the preoperative value within the first 7 postoperative days or until the end of laboratory controls.

All analyses were conducted on patients who had information on all of the variables used for the propensity score matching, and no imputation of missing data was intended. As a result, we disqualified patients whose data on any of the variables for the matching were lacking. All tests were two-sided, and statistical significance was considered with a *p* value less than 0.05. The statistical analyses were performed using R 4.2.2 GUI 1.79 Big Sur ARM build (R basis for statistical calculation; Vienna University of Economics and Business, Vienna, Austria) using the packages “tableone”, “MatchIt”, “Cobalt”, “tidyverse” and “gplot2”.

## 3. Results

A total of 769 subjects were included in the analysis from our registry in the unmatched cohort. The selection period was between 1/2021 and 10/2022. The majority, *n* = 565, of the subjects were monitored with invasive blood pressure measurement alone. N = 204 subjects were additionally monitored with the HPI system. The two groups in the unmatched cohort differed in several characteristics (Table 1, Table 2 and Table 3).

Patients in the non-HPI group had a higher status in ASA classification (*p* < 0.001). The frequency of emergency surgery was also higher in the non-HPI group (131 of 565 (23.2%) vs. 2 of 204 (1.0%), *p* < 0.001). Co-morbidities were comparable between groups, e.g., chronic obstructive pulmonary disease, diabetes mellitus or arterial hypertension. Antihypertensive medication differed slightly in the intake of calcium channel blockers (225 of 565 (21.8%) vs. 27 of 204 (13.2%), *p* = 0.011) but was comparable in other antihypertensive drug groups, e.g., ACE inhibitors, beta blockers, diuretics and AT1 receptor antagonists.

Preoperative concentration of hemoglobin also differed slightly and was higher in the HPI group (13.0 g/dL, (IQR 11.38, 14.10)) than in non-HPI subjects (12.5 g/dL, (IQR 10.10, 14.10), *p* = 0.029). The surgical approach as well as the duration of surgery were significantly different between the two groups. An epidural catheter was more often applied in HPI subjects (169 of 294 (82.8%)) than in the non-HPI group (185 of 565 (32.7%), *p* < 0.001).

**Figure 1:**
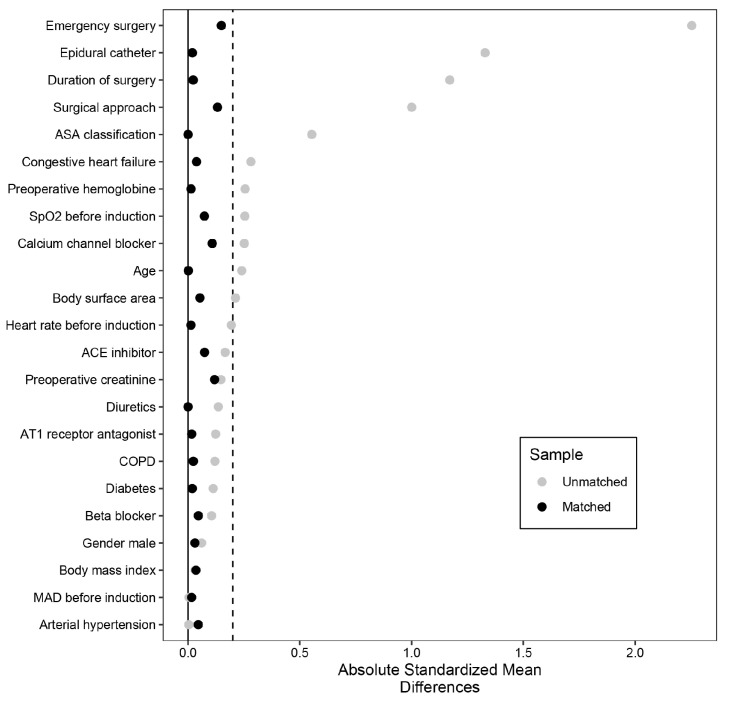
Covariate balance. Absolute standardized means were higher than 0.2 in the unmatched cohort in many variables and were shown to be below 0.2 after matching. ASA, American Society of Anesthesiologists; ACE, angiotensin-converting-enzyme; AT1, Angiotensin II receptor type 1. COPD, chronic obstructive pulmonary disease; MAD, mean arterial pressure.

Absolute standardized mean differences were higher than 0.2 in many variables (Figure 1).

Concerning the primary endpoint, there was a higher TWA with threshold below 65 mmHg (0.200 (IQR 0.040, 0.540) vs. 0.05 (IQR 0.010, 0.200), *p* < 0.001) in the full, unmatched cohort. The same pattern was also seen in cumulative duration of hypotension per patient (5.00 (IQR 1.00, 13.0) vs. 3.00 (IQR 1.00, 10.0) minutes, *p* = 0.002) and TWA below 60 mmHg.

In HPI-monitored subjects there was an average of 8 events with an HPI equal or greater than 85, while hypotensive events were seen with an average number of 2.00 (IQR 0.00, 4.00) (Table 4).

After propensity score matching for all of the variables listed in Table 1 and Table 2 there were no longer statistically significant differences between non-HPI and HPI (*n* = 136 in each group) subjects for demographic and intraoperative data (Table 5 and Table 6).

Absolute standardized means were shown below 0.2 (Figure 1). The same pattern was seen for the administration of intraoperative medication and fluid (Table 7), except for the administered volume of crystalloid fluid (4000 (IQR 3000, 7000) vs. 6000 (IQR 4000, 7500) mL, *p* = 0.001). 

In addition, the cumulative dose of Norepinephrine was without significant difference between non-HPI and HPI-treated patients (1.76 (IQR 0.69, 3.18) vs. 1.99 (0.87, 3.53) mg, *p* = 0.429) while the amount of applied crystalloid volume was higher in the HPI group (6000 (IQR 4000, 7500) vs. 4000 (IQR 3000, 7000) mL in the non-HPI group, *p* = 0.001).

The time-weighted average of hypotension below 65 mmHg as the primary endpoint in the matched cohort was 0.180 mmHg (IQR 0.060, 0.410) in the non-HPI group vs. 0.070 mmHg (IQR 0.020, 0.240) in the HPI group (*p* < 0.001, Table 8).

Moreover, secondary endpoints also showed significant differences between both groups in the matched cohort; the number of hypotensive events was 3.00 (IQR 1.00, 5.25) in the non-HPI group vs. 2.00 (IQR 1.00, 4.00) in HPI subjects (*p* = 0.002; Table 8) and the cumulative duration of hypotension below 65 mmHg was higher in non-HPI patients (7.00 (IQR 2.00, 15.3) vs. 3.00 (IQR 1.00, 10.0) minutes, *p* = 0.001).

**Figure 2:**
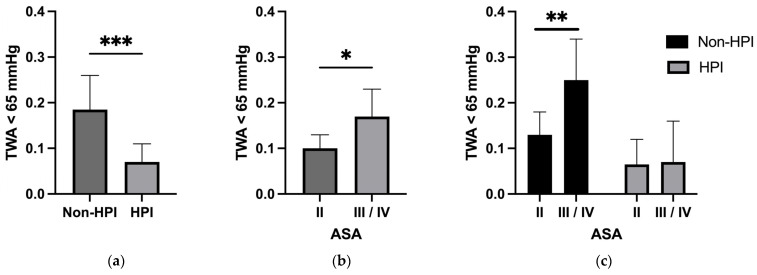
TWA below 65 mmHg in (**a**) non-HPI and HPI-treated subjects in matched cohort; (**b**) for all patients with ASA status II vs. III/IV in matched cohort; (**c**) for non-HPI and HPI subjects considering ASA status II vs. III/IV in matched cohort. TWA, time weighted average; ASA, American Society of Anesthesiologists; HPI, Hypotension Prediction index; * *p* < 0.05; ** *p* < 0.01; *** *p* < 0.001.

In HPI-monitored subjects there was an average of 8 alarms with HPI values equal to or greater than 85, while hypotensive events were seen only with an average number of 2.00 (IQR 1.00, 4.00) (Table 8; Figure 2a).

While ASA physical status was shown to be associated with intraoperative hypotension [14] we considered incidences of hypotension with the ASA status of the matched cohort and could demonstrate higher TWA values below 65 mmHg in patients with ASA classification III/IV (0.170 (IQR 0.020, 0.250)) compared with patients classified as ASA status II (0.100 (IQR 0.035, 0.365), *p* = 0.02, Figure 2b). Interestingly, stratification of the intervention groups revealed significant differences for the non-HPI group, which had more than twice the TWA values of the HPI group (*p* = 0.010); however, patients with HPI monitoring showed similar TWA values in different ASA groups, indicating an advantageous effect of hypotension prevention using HPI in patients at higher risk (Figure 2c).

Regarding postoperative data in the matched cohort, there were no significant differences in terms of postoperative hemoglobin levels, length of stay in the ICU or in terms of postoperative maximum serum creatinine levels. However, there was a trend of higher incidences of postoperative acute renal injury in non-HPI patients (Table 9).

## 4. Discussion

In this study, we compared two groups with different hemodynamic monitoring methods, i.e., HPI vs. standard treatment with arterial waveform analysis alone in an unmatched and propensity-score-matched cohort. The presented data show that use of HPI reduced hypotension during noncardiac surgery in both a matched and unmatched cohort. This was true for time-weighted average and relative time in hypotension < 65 mmHg as well as for absolute numbers of hypotensive events during the monitoring time. In addition, patients with ASA status III/IV were identified with higher TWA values below 65 mmHg while HPI monitoring prevented these differences and showed significantly lower values even in higher ASA classes. Finally, postoperative renal failure, although not significantly different, tended to occur more frequently in the non-HPI group.

In line with the results of our work, Wijnberge et al. [14] as well as Schneck et al. [15] showed in blinded, randomized controlled trials that intraoperative hypotension could be significantly reduced under monitoring with HPI compared with a control group. Wijnberge et al. demonstrated a time-weighted average of 0.10 mmHg in the HPI group while the control group had a time-weighted average of 0.40 mmHg. 

In our study we could show a median TWA of 0.180 mmHg in the non-HPI group, in contrast with the former study. The lower value of the TWA in our control group compared with the results of former study groups could be due to the clinicians’ learning effects and awareness of perioperative hypotension on the one hand, and, on the other hand, our internal goal to keep the mean arterial pressure of all patients above a minimum of 65 mmHg. 

Given the fact that we are not aware of standard operating procedures in that center, hypotension might have been handled more liberally there—especially in ASA II patients, whose proportion in the study was significantly higher at 80% compared with the 39% in our cohort [14]. 

Another influence of the different TWAs in the study control groups may be the use of GDTs. Schneck et al., for example, showed a significant reduction in intraoperative hypotension for patients treated with HPI monitoring in comparison with an historical control group. In contrast with our study, a possible influence on their results might been the lack of an applied treatment protocol in the control group. The positive effects of standard operation procedures and GDT protocols have been reported in different clinical settings [16,17,18]. 

In contrast with the aforementioned studies and our data, Maheshwari et al. could not show a significant difference when comparing an HPI-guided group with an unguided group [12]. They presented comparable values in both groups for a time-weighted average of 0.14 mmHg and a time-weighted mean arterial pressure >83 mmHg (84.9 vs. 83.4 mmHg, *p* = 0.193). In their study, clinicians were asked to avoid an MAP of less than 65 mmHg, and, under this observation, clinicians may well have initiated aggressive strategies to reduce hypotension under clinical supervision, which is common in a prospective study. A bias effect cannot be excluded here which is called the Hawthorne effect and the actual effects could thus remain masked [19].

As a result of these inconsistencies, and in order to incorporate more reliable data on the population level, we employed propensity score matching in the current work. This method allows the estimation of causal intervention effects in non-randomized research by minimizing the selection bias between interventional groups [13]. By using this method, we created a dataset with low bias as well as significant demographic, anesthetic and surgical information. While enlarging the caliper size for this statistical method increases the size of the resulting comparison groups, it also reduces the quality and thus the transferability of the results. Therefore, we decided to keep the quality of the matching as high as possible with a caliper size of 0.1, which at the same time should also have increased the stability and transferability of the presented results.

In addition to ASA classification, surgical approach, and duration of the procedure, the use of an epidural catheter and the declaration of the procedure as an emergency procedure were most impressive when considering the differences and corresponding SMDs in the unmatched cohort. While a sympatholytic effect with consecutive hypotension by epidural anesthesia seems reasonable, the high rate of emergency interventions in the non-HPI group of the unmatched cohort is an important point to demonstrate the importance of matching when evaluating such data. Furthermore, the clinicians in charge of anesthesia only had the information to keep the MAP above 65 mmHg or to follow the personalized treatment protocol. None of the anesthesiologists knew that the data were to be used in this study.

Intraoperative hypotension in patients undergoing noncardiac surgery remains an important risk for adverse events. Ahuja et al. [5] have recently demonstrated that the risk for myocardial, and especially kidney injury, increases with duration and severity of intraoperative hypotension. In addition, Mathis et al. [8] have shown that more severe comorbidities increase the risk of postoperative complications and that patients with higher risk factors may benefit to a greater extent from advanced hemodynamic monitoring. Südfeld et al. [20] have also shown that patients with ASA physical status IV in particular have a significantly higher risk for early intraoperative hypotension. Our data show that TWA values in patients with ASA status III/IV were only half as high under extended hemodynamic monitoring with HPI as under invasive pressure measurement alone. 

Futier et al. [7] have already demonstrated, in a randomized controlled trial and with a cohort of about 59% ASA status III or higher, that maintaining a higher MAP during abdominal surgery reduces the risk of postoperative organ dysfunction by about 25%, suggesting that the association between hypotension and organ injury is at least partially causal and therefore potentially amenable to intervention. This indicates an advantageous effect of hypotension prevention using HPI in patients at higher risk.

This effect may finally translate into a higher incidence of postoperative kidney failure in non HPI-patients, which was observed in a trend in our study. However, this outcome was not the primary aim, therefore the number was too small to detect significant differences. 

Nevertheless, there is increasing debate in the current literature as to whether the use of arterial pressure alarms alone would be equivalent to the HPI system. Therefore, the presumed added value of the Hypotension Prediction Index over the MAP has been found through steady discussion, with Mulder et al. [21] arguing that setting an alarm at an MAP of approximately 70 to 75 mmHg might yield a prediction of intraoperative hypotension comparable to the Hypotension Prediction Index.

In this study, an MAP of greater than 65 mmhg was targeted in all patients in both the HPI and non-HPI groups according to in-house standards. In addition, in patients with relevant corresponding comorbidities and expected autoregulatory mechanisms, MAP was kept within individual higher limits.

This strict GDT strategy may have resulted in the lower value of TWA below 65 mmHg of 0.180 mmHg in the non-HPI group compared with the higher values in other studies [14,22,23,24,25]. Nevertheless, we could show a further reduction of these already low TWAs under HPI monitoring. This is true in the group of ASA II as well as ASA III/IV patients and results in TWA values that are comparable to data from the EU-HYPROTECT study [26]. At the same time, administration of crystalloid fluid was higher in the HPI group compared with the non-HPI group. This higher volume application in the HPI group of our matched cohort might also be the result of a strict adherence to the strategy specified in the GDT protocol. Anesthesiologists in the control group might have tended to increase the vasopressor when hypotension occurred, because extended hemodynamic variables were not available for the decision of needed treatment.

Interestingly, our data show a median incidence of eight HPI alarms per patient in the HPI monitored group while the median number of hypotensive events was only two per patient. In purely arithmetic terms, this resulted in six prevented hypotension events per patient or 816 prevented hypotension events in the HPI group. On the other hand, patients with invasive arterial monitoring alone experienced 1.5-fold more hypotensive events compared with patients with HPI monitoring. Thus, it can be argued that, while clinical standards and MAP alarms are certainly suitable for the prevention of hypotension to a high degree, HPI provides additional reassurance and also clues to possible causes for individualized treatment.

Nevertheless, due to its monocentric nature, one limitation is that our results cannot be generalized to other clinical centers. Moreover, the arterial line was placed after anesthesia induction. For upcoming studies, a different time of placement could be considered, as up to one third of intraoperative hypotension events occur shortly after induction [2,27,28]. Finally, the accuracy of the HPI algorithm is highly dependent on the quality of the arterial waveform signal, therefore accurate waveform signals are mandatory [29,30]. Moreover, HPI is able to predict hypotension in the presence of vasoactive and inotropic substances, while surgical causes of hypotension (e.g., clamping of the inferior vena cava) cannot be predicted due to the lack of physiological prodromal changes in the arterial waveform. 

This study employed a retrospective but propensity-score-matched design. This method allows the estimation of causal intervention effects in non-randomized research by minimizing the selection bias between interventional groups. This bias might have been an influence on the previous studies with inconsistent results. 

Furthermore, in this trial, the depth of anesthesia was measured in contrast with former studies, e.g., Wijnberge et al. [14]. In a recent randomized controlled trial, a significant reduction in Norepinephrine dose was observed using electroencephalographic monitoring in patients under anesthesia [31]. However, the data of this study seem to reflect a setting that is transferable to real clinical conditions.

Within the framework of this aspect, a perspective view may also be ventured. While the results of our work reflect the isolated observation and reaction to hemodynamic variables, the benefit of a machine-learning model, which also includes other variables such as volume inflow and outflow, depth of anesthesia, applied vasopressors or inotropics in its analysis, should be even greater. The assessment of such variables and the derivation of corresponding therapy suggestions in the sense of a GDT on the basis of machine learning should be the goal of future developments in this field.

## 5. Conclusions

We showed a significant reduction in incidence and duration as well as in the severity of intraoperative hypotension for patients treated with the HPI compared with arterial waveform analysis alone. Furthermore, patients classified as ASA III or IV were seen to benefit most from the advanced monitoring. Further investigation on larger study populations is needed to detect differences between other outcome parameters.

## Figures and Tables

**Table 1:** Demographical data for unmatched cohort.

Parameter	Non-HPI *n* = 565	HPI *n* = 204	*p* Value	SMD
Age in years, median (IQR)	70.0 (61.0, 77.0)	68.0 (60.0, 74.0)	0.007	0.237
Gender male, *n* (%)	352 (62.3)	133 (65.2)	0.516	0.060
Body surface area in m^2^, median (IQR)	1.96 (1.80, 2.12)	2.04 (1.88, 2.17)	0.002	0.205
Body mass index in kg/m^2^, median (IQR)	27.0 (24.0, 30.5)	27.8 (24.8, 30.4)	0.161	0.028
ASA classification, *n* (%)			<0.001	0.576
I	0 (0)	0 (0)		
II	118 (20.9)	94 (46.1)		
III	384 (68.0)	101 (49.5)		
IV	63 (9.4)	9 (4.4)		
Emergency surgery, *n* (%)	131 (23.2)	2 (1.0)	<0.001	0.725
Congestive heart failure, *n* (%)	53 (9.4)	8 (3.9)	0.020	0.220
Chronic obstructive pulmonary disease, *n* (%)	82 (14.5)	22 (10.8)	0.224	0.112
Diabetes, *n* (%)	127 (22.5)	37 (18.1)	0.231	0.108
Arterial hypertension, *n* (%)	361 (63.9)	130 (63.7)	1.000	0.004
ACE inhibitor, *n* (%)	148 (26.2)	40 (19.6)	0.075	0.157
Beta blocker, *n* (%)	225 (39.8)	71 (34.8)	0.238	0.104
Calcium channel blocker, *n* (%)	123 (21.8)	27 (13.2)	0.011	0.226
Diuretics, *n* (%)	132 (32.4)	37 (18.1)	0.148	0.129
AT1 receptor antagonist, *n* (%)	124 (21.9)	56 (27.5)	0.135	0.128
Preoperative hemoglobin concentration in g/dL, median (IQR)	12.5 (10.1, 14.1)	13.0 (11.4, 14.1)	0.029	0.222
Preoperative creatinine in mg/dL, median (IQR)	1.00 (0.80, 1.20)	0.90 (0.80, 1.10)	0.066	0.139

Quantitative variables are presented as means and standard deviation (SD) in case of normal distribution, otherwise, data are presented as median and interquartile range (IQR). HPI, hypotension prediction index; SMD, standardized mean difference; ASA, American Society of Anesthesiologists; ACE, angiotensin-converting-enzyme; AT1, Angiotensin II receptor type 1.

**Table 2:** Intraoperative data for unmatched cohort.

Parameter	Non-HPI *n* = 565	HPI *n* = 204	*p* Value	SMD
Surgical approach, *n* (%)			<0.001	0.839
Laparoscopy	97 (17.2)	63 (30.9)		
Laparotomy	218 (38.6)	119 (58.3)		
Combined	37 (6.5)	8 (3.9)		
Other	213 (37.7)	14 (6.9)		
Duration of surgery in min, median (IQR)	143 (99, 210)	311 (228, 392)	<0.001	1.250
Epidural catheter, *n* (%)	185 (32.7)	169 (82.8)	<0.001	1.177
Mean arterial pressure at induction in mmHg, median (IQR)	95.0 (86.0, 105.0)	96.0 (87.8, 103.3)	0.829	0.005
Heart rate before induction in bpm, median (IQR)	76.0 (68.0, 90.0)	76.5 (68.0, 87.0)	0.341	0.166
Oxygen saturation before in induction in %, median (IQR)	97.0 (95.0, 99.0)	97.0 (96.0, 99.0)	0.167	0.203

Quantitative variables are presented as means and standard deviation (SD) in case of normal distribution, otherwise, data are presented as median and interquartile range (IQR). HPI, hypotension prediction index; SMD, standardized mean difference.

**Table 3:** Intraoperative medication and fluid administration for unmatched cohort.

Parameter	Non-HPI *n* = 565	HPI *n* = 204	*p* Value	SMD
Mean minimal alveolar concentration in balanced anesthesia, median (IQR)	0.93 (0.84, 1.03)	0.97 (0.90, 1.04)	0.005	0.082
Cumulative dose of Ropivacaine in epidural application in mg, median (IQR)	142 (112, 187)	225 (155, 262)	<0.001	0.905
Cumulative dose of Norepinephrinein mg, median (IQR)	0.72 (0.20, 1.93)	2.24 (0.94, 3.96)	<0.001	0.393
Cumulative dose of Dobutaminein mg, median (IQR)	30.8 (11.1, 47.1)	45.0 (22.7, 82.4)	0.161	0.138
Amount of crystalloid fluid in mL, median (IQR)	3000 (2000, 4500)	6000 (5000, 8000)	<0.001	0.982
Amount of colloid fluid in mL, median (IQR)	500 (500, 1000)	750 (500, 1000)	0.272	0.128
Estimated blood loss in mL, median (IQR)	455 (200, 700)	600 (300, 900)	<0.001	0.298

Quantitative variables are presented as means and standard deviation (SD) in case of normal distribution, otherwise, data are presented as median and interquartile range (IQR). HPI, hypotension prediction index; SMD, standardized mean difference.

**Table 4:** Endpoints for unmatched cohort.

Primary Endpoints	Non-HPI *n* = 565	HPI *n* = 204	*p* Value	SMD
Time-weighted average (MAP < 65 mmHg) in mmHg, median (IQR)	0.200 (0.040, 0.540)	0.050 (0.010, 0.200)	<0.001	0.384
Secondary Endpoints				
Number of hypotensive events (MAP < 65 mmHg) per patient, median (IQR)	2.00 (1.00, 5.00)	2.00 (0.00, 4.00)	0.097	0.022
Cumulative duration of hypotension (MAP < 65 mmHg) per patient in minutes, median (IQR)	5.00 (1.00, 13.0)	3.00 (1.00, 10.0)	0.002	0.147
Time-weighted average (MAP < 60 mmHg) in mmHg, median (IQR)	0.040 (0.000, 0.160)	0.010 (0.000, 0.050)	<0.001	0.278
Time-weighted average (MAP < 55 mmHg) in mmHg, median (IQR)	0.000 (0.000, 0.030)	0.000 (0.000, 0.020)	0.022	0.164
Number of events HPI > 85, median (IQR)	NA	8.00 (4.75, 14.3)	NA	NA

Quantitative variables are presented as means and standard deviation (SD) in case of normal distribution, otherwise, data are presented as median and interquartile range (IQR). HPI, hypotension prediction index; SMD, standardized mean difference; MAP, mean arterial pressure; NA, not applicable.

**Table 5:** Demographical data for matched cohort.

Parameter	Non-HPI *n* = 136	HPI *n* = 136	*p* Value	SMD
Age in years, median (IQR)	67.0 (59.0, 75.0)	68.00 (60.0, 75.0)	0.725	0.001
Gender male, *n* (%)	85 (62.5)	83 (61.0)	0.901	0.031
Body surface area in m^2^, median (IQR)	2.00 (1.86, 2.10)	1.99 (1.88, 2.11)	0.671	0.053
Body mass index in kg/m^2^, median (IQR)	27.2 (23.9, 31.3)	27.05 (24.8, 30.0)	0.948	0.035
ASA classification, *n* (%)			0.206	<0.001
I	0 (0)	0 (0)		
II	51 (37.5)	56 (41.2)		
III	82 (60.3)	72 (52.9)		
IV	3 (2.2)	8 (5.9)		
Emergency surgery, *n* (%)	4 (2.9)	2 (1.5)	0.680	0.149
Congestive heart failure, *n* (%)	5 (3.7)	6 (4.4)	1.000	0.038
Chronic obstructive pulmonary disease, *n* (%)	14 (10.3)	13 (9.6)	1.000	0.024
Diabetes, *n* (%)	21 (15.4)	22 (16.2)	1.000	0.019
Arterial hypertension, *n* (%)	87 (64.0)	84 (61.8)	0.802	0.046
ACE inhibitor, *n* (%)	24 (17.6)	28 (20.6)	0.644	0.074
Beta blocker, *n* (%)	49 (36.0)	46 (33.8)	0.799	0.046
Calcium channel blocker, *n* (%)	22 (16.2)	17 (12.5)	0.489	0.109
Diuretics, *n* (%)	25 (18.4)	25 (18.4)	1.000	<0.001
AT1 receptor antagonist, *n* (%)	38 (27.9)	37 (27.2)	1.000	0.017
Preoperative hemoglobin concentration in g/dL, median (IQR)	12.9 (11.3, 14.4)	13.00 (11.3, 14.3)	0.907	0.014
Preoperative creatinine in mg/dL, median (IQR)	0.900 (0.800, 1.10)	0.900 (0.800, 1.10)	0.893	0.119

Quantitative variables are presented as means and standard deviation (SD) in case of normal distribution, otherwise, data are presented as median and interquartile range (IQR). HPI, hypotension prediction index; SMD, standardized mean difference; ASA, American Society of Anesthesiologists; ACE, angiotensin-converting-enzyme; AT1, Angiotensin II receptor type 1.

**Table 6:** Intraoperative data for matched cohort.

Parameter	Non-HPI *n* = 136	HPI *n* = 136	*p* Value	SMD
Surgical approach, *n* (%)			0.065	0.132
Laparoscopy	35 (25.7)	32 (23.5)		
Laparotomy	67 (49.3)	84 (61.8)		
Combined	17 (12.5)	6 (4.4)		
Other	17 (12.5)	14 (10.3)		
Duration of surgery in min, median (IQR)	255 (166, 342)	266 (174, 332)	0.767	0.023
Epidural catheter, *n* (%)	102 (75.0)	101 (74.3)	1.000	0.020
Mean arterial pressure at induction in mmHg, median (IQR)	94.0 (86.0, 106)	95.5 (87.5, 104)	0.945	0.016
Heart rate before induction in bpm, median (IQR)	75.0 (66.0, 85.0)	76.5 (68.0, 87.0)	0.688	0.013
Oxygen saturation before in induction in %, median (IQR)	98.0 (96.0, 99.0)	98.0 (96.0, 99.0)	0.584	0.073

Quantitative variables are presented as means and standard deviation (SD) in case of normal distribution, otherwise, data are presented as median and interquartile range (IQR). HPI, hypotension prediction index; SMD, standardized mean difference.

**Table 7:** Intraoperative medication and fluid administration for matched cohort.

Parameter	Non-HPI *n* = 136	HPI *n* = 136	*p* Value	SMD
Mean minimal alveolar concentration in balanced anesthesia, median (IQR)	0.960 (0.880, 1.07)	0.970 (0.870, 1.03)	0.813	0.090
Cumulative dose of Ropivacaine in epidural application in mg, median (IQR)	187 (121, 225)	187 (150, 255)	0.128	0.188
Cumulative dose of Norepinephrine in mg, median (IQR)	1.76 (0.69, 3.18)	1.99 (0.87, 3.53)	0.429	0.033
Cumulative dose of Dobutamine in mg, median (IQR)	43.6 (29.8, 58.4)	48.2 (27.4, 74.8)	0.741	0.050
Amount of crystalloid fluid in mL, median (IQR)	4000 (3000, 7000)	6000 (4000, 7500)	0.001	0.281
Amount of colloid fluid in mL, median (IQR)	1000 (500, 1000)	500.0 (500, 100)	0.709	0.104
Estimated blood loss in mL, median (IQR)	480 (200, 900)	550 (300, 900)	0.115	0.045

Quantitative variables are presented as means and standard deviation (SD) in case of normal distribution, otherwise, data are presented as median and interquartile range (IQR). HPI, hypotension prediction index; SMD, standardized mean difference.

**Table 8:** Endpoints for matched cohort.

Primary Endpoints	Non-HPI *n* = 136	HPI *n* = 136	*p* Value	SMD
Time-weighted average (MAD < 65 mmHg) in mmHg, median (IQR)	0.180 (0.060, 0.410)	0.070 (0.020, 0.240)	<0.001	0.243
Secondary endpoints				
Number of hypotensive events (MAD < 65 mmHg) per patient, median (IQR)	3.00 (1.00, 5.25)	2.00 (1.00, 4.00)	0.002	0.338
Cumulative duration of hypotension (MAD < 65 mmHg) per patient in minutes, median (IQR)	7.00 (2.00, 15.3)	3.00 (1.00, 10.0)	0.001	0.279
Time-weighted average (MAD < 60 mmHg) in mmHg, median (IQR)	0.040 (0.000, 0.120)	0.010 (0.000, 0.060)	0.005	0.153
Time-weighted average (MAD < 55 mmHg) in mmHg, median (IQR)	0.010 (0.000, 0.030)	0.000 (0.000, 0.020)	0.046	0.053
Number of HPI alarms > 85, median (IQR)	NA	8.00 (4.00, 12.3)	NA	NA

Quantitative variables are presented as means and standard deviation (SD) in case of normal distribution, otherwise, data are presented as median and interquartile range (IQR). HPI, hypotension prediction index; SMD, standardized mean difference; MAP, mean arterial pressure; NA, not applicable.

**Table 9:** Postoperative data for matched cohort.

Parameter	Non-HPI *n* = 136	HPI *n* = 136	*p* Value	SMD
Postoperative hemoglobin in g/dL, median (IQR)	10.2 (8.70, 11.7)	9.70 (8.60, 11.2)	0.118	0.186
ICU submissions, *n* (%)	91 (66.9)	113 (83.1)	0.003	0.380
Length of stay in ICU in hours, median (IQR)	70.0 (25.0, 129.5)	91.0 (47.0, 113.0)	0.161	0.065
Death in ICU, *n* (%)	6 (6.5)	5 (4.4)	0.728	0.091
Acute renal failure, *n* (%)	20 (14.7)	11 (8.1)	0.127	0.209
Postoperative maximum creatinine, median (IQR)	1.00 (0.80, 1.30)	1.00 (0.80, 1.30)	0.795	0.030
Need for renal replacement therapy in ICU, *n* (%)	3 (3.2)	4 (3.5)	1.000	0.016

Quantitative variables are presented as means and standard deviation (SD) in case of normal distribution, otherwise, data are presented as median and interquartile range (IQR). HPI, hypotension prediction index; SMD, standardized mean difference; ICU, intensive care unit.

## Data Availability

The data presented in this study are available on request from the corresponding author. The data are not publicly available due to ethical restrictions.
